# Supplementary material for: An Experimental Model of Proton-Beam-Induced Radiation Dermatitis In Vivo
Source: Int J Mol Sci. 2023 Nov 15;24(22):16373. doi: 10.3390/ijms242216373 (PMC10671732; doi:10.3390/ijms242216373)
Supplement: Supplementary file 1 [file ijms-24-16373-s001.zip › ijms-2670050-supplementary.pdf]

# Supplementary materials

**Table S1:** Effect of proton irradiation on the morphological composition of peripheral blood in white male SHK mice in RD modeling. The experimental data were presented as the M ± SD ($10^9$/L) and were analyzed using a Student's t-test (* p < 0.05 vs Initial, ** p < 0.01 vs Initial).

| Days after irradiation | Test group | | | | | | | |
|---|---|---|---|---|---|---|---|---|
| | **CND** | **ND30** | **ND40** | **CD** | **D30** | **D40** | **CChD** | **ChD50** |
| White blood cell count (WBC), $10^9$/L | | | | | | | | |
| Initial | 12.89 ± 4.71 | | | | | | | |
| 7th day | 11.65 ± 0.73 | 9.39 ± 2.03 | 12.18 ± 0.98 | 7.39 ± 0.96 | 11.59 ± 3.55 | 4.60 ± 1.56 | 12.19 ± 1.26 | 17.54 ± 5.66 |
| 21st day | 9.16 ± 0.54 | 13.90 ± 1.06 | 12.19 ± 4.66 | 13.27 ± 1.21 | 7.91 ± 2.60 | 15.81 ± 5.43 | 18.16 ± 3.16 | 24.03 ± 8.34 |
| 70th day | 8.55 ± 0.61 | 14.44 ± 3.55 | 12.91 ± 5.46 | 9.50 ± 5.94 | 14.59 ± 5.67 | 8.27 ± 3.80 | 17.71 ± 3.39 | 14.30 ± 4.16 |
| Lymphocytes count (Lym), $10^9$/L | | | | | | | | |
| Initial | 7.13 ± 2.39 | | | | | | | |
| 7th day | 8.32 ± 3.57 | 6.43 ± 1.84 | 6.41 ± 0.38 | 4.51 ± 1.87 | 3.49 ± 1.27 | 7.19 ± 1.81 | 6.96 ± 0.09 | 9.96 ± 2.94 |
| 21st day | 5.53 ± 2.74 | 6.44 ± 2.26 | 7.46 ± 2.87 | 7.41 ± 2.09 | 9.45 ± 2.90 | 4.99 ± 1.67 | 9.69 ± 1.04 | 10.21 ± 3.29 |
| 70th day | 5.60 ± 3.80 | 8.81 ± 2.63 | 6.77 ± 2.43 | 6.11 ± 4.06 | 4.73 ± 2.12 | 7.65 ± 2.61 | 7.65 ± 1.03 | 8.03 ± 2.77 |
| Granulocytes count (Gran), $10^9$/L | | | | | | | | |
| Initial | 3.67 ± 2.14 | | | | | | | |
| 7th day | 2.03 ± 0.92 | 2.00 ± 0.26 | 4.07 ± 0.90 | 1.74 ± 0.23 | 1.77 ± 0.21 | 2.72 ± 1.21 | 2.89 ± 0.29 | 4.71 ± 2.07 |
| 21st day | 2.23 ± 0.33 | 4.79 ± 0.63 | 2.93 ± 1.15 | 3.70 ± 0.91 | 5.55 ± 1.75 | 1.97 ± 0.76 | 5.00 ± 0.98 | 8.61 ± 4.31 |
| 70th day | 1.98 ± 0.22 | 3.48 ± 0.70 | 4.74 ± 2.84 | 2.27 ± 1.37 | 2.23 ± 1.18 | 3.94 ± 1.54 | 6.23 ± 1.93 | 3.92 ± 1.4 |
| Platelets count (PLT), $10^9$/L | | | | | | | | |
| Initial | 406 ± 78 | | | | | | | |
| 7-th day | 518 ± 42 | 552 ± 107 | 373 ± 67 | 776 ± 113 | 573 ± 75 | 412 ± 129 | 896 ± 215 | 1059 ± 223 * |

| | | | | | | | | |
|---|---|---|---|---|---|---|---|---|
| 21-th day | 665 ± 73 | 339 ± 58 | 642 ± 103 | 607 ± 54 | 338± 35 | 537 ± 147 | 994 ± 56 ** | 1049 ± 352 |
| 70-th day | 640 ± 138 | 491 ± 96 | 406 ± 157 | 585 ± 25 | 640 ± 267 | 419 ± 140 | 895 ± 3 ** | 1011 ± 349 |
